# Iron in ventricular remodeling and aneurysms post-myocardial infarction

**DOI:** 10.1515/med-2024-1109

**Published:** 2024-12-11

**Authors:** Zuoyan Wang, Xiang Ding, Jingyu Pan, Xueyan Wang, Jieming Lin, Xinzhu Wang

**Affiliations:** Department of Cardiology, Beijing Shijitan Hospital, Capital Medical University, No. 10 Tieyi Road, Haidian District, Beijing, 100038, China; Department of Clinical Medicine, Capital Medical University, Beijing, China

**Keywords:** myocardial infarction, ventricular remodeling, left ventricular aneurysm, iron metabolism, oxidative stress, ferroptosis

## Abstract

**Background:**

Post-myocardial infarction (MI) complications, including ventricular remodeling (VR) and left ventricular aneurysm (LVA) formation, significantly affect patient prognosis and quality of life. Both iron overload and deficiency play critical roles in these pathological processes.

**Objectives:**

This review aims to explore the mechanisms linking abnormal iron metabolism with post-MI VR and LVA formation and to highlight therapeutic strategies that regulate iron levels to mitigate adverse cardiac remodeling.

**Methods:**

The review analyzes existing clinical and experimental research on the role of iron metabolism in post-MI complications. It focuses on iron overload, oxidative stress, ferroptosis, and the impact of iron deficiency on mitochondrial function, energy metabolism, and cardiomyocyte repair.

**Results:**

Iron overload exacerbates myocardial injury through oxidative stress, ferroptosis, and inflammation, leading to fibrosis and ventricular dilation. In contrast, iron-deficiency impairs mitochondrial function, energy metabolism, and cardiomyocyte repair, further contributing to adverse remodeling outcomes. Therapeutic strategies such as iron chelators, ferroptosis inhibitors, and iron supplementation are potential interventions for mitigating adverse remodeling.

**Conclusion:**

Abnormal iron metabolism, both overload and deficiency, plays a critical role in post-MI complications. Therapeutic strategies targeting iron levels hold promise for reducing adverse cardiac remodeling and improving patient outcomes after MI.

## 1 Introduction

Acute ST-segment elevation myocardial infarction (STEMI) is the most severe form of coronary artery disease, typically accompanied by significant myocardial injury and structural remodeling [1]. Post-myocardial infarction (MI) ventricular remodeling (VR) and aneurysm formation are common complications, severely affecting patient prognosis and quality of life [2]. Ventricular remodeling refers to changes in heart morphology, size, and function after MI, while aneurysm formation represents an extreme form of VR, characterized by localized ventricular wall dilation, increasing the risk of heart failure and arrhythmias [3].

In recent years, increasing research has focused on the role of serum iron levels in post-MI. Iron, an essential trace element, plays a crucial role in oxygen transport, energy metabolism, and DNA synthesis. However, an imbalance in iron metabolism, whether iron overload or deficiency, can negatively impact cardiac health [4]. Studies have shown that abnormal serum iron levels are closely associated with VR and aneurysm formation after MI [5,6]. Excess iron ions can exacerbate myocardial injury and fibrosis by promoting oxidative stress and inflammatory responses [7], thereby accelerating VR. Additionally, iron can influence the activity of cardiac fibroblasts, promoting collagen synthesis and deposition [8], which leads to ventricular wall stiffening and dilation. On the other hand, iron deficiency may impair energy metabolism and the repair capacity of cardiomyocytes, further affecting the process and outcome of VR [9].

To better understand the role of serum iron levels in VR and aneurysm formation following STEMI, this article will comprehensively analyze existing clinical research findings. It will explore how abnormalities in iron metabolism affect changes in cardiac structure and function and propose potential therapeutic strategies.

## 2 Characteristics of iron metabolism in acute myocardial infarction (AMI)

AMI is a severe cardiovascular event caused by the sudden obstruction of a coronary artery, leading to interrupted blood supply to the myocardium, resulting in hypoxia and necrosis of myocardial cells. In this pathological state, the process of iron metabolism undergoes significant changes. Under normal conditions, iron is absorbed, transported, stored, and utilized through tightly regulated metabolic pathways. However, the occurrence of AMI and the subsequent biological responses profoundly affect the process of iron metabolism.

Iron absorption primarily occurs in the small intestine, especially in the duodenum. Dietary iron is classified into two types: heme iron and non-heme iron. Heme iron has higher bioavailability and is less affected by inhibitory factors, being directly absorbed by the intestine [10]. Non-heme iron, such as ferrous sulfate, is mainly absorbed with the help of divalent metal transporter 1 (DMT1) across the apical membrane of intestinal epithelial cells and then transported into the circulatory system through ferroportin (FPN) [11]. In the plasma, transferrin (Tf) binds to the iron released into circulation, forming a transferrin-iron complex (Tf-Fe) that transports iron through the bloodstream to target cells requiring iron [12]. On the membrane of these target cells, transferrin receptor 1 (TfR1) specifically binds to the transferrin-iron complex, forming a transferrin-iron-transferrin receptor complex, which is then internalized through endocytosis, allowing iron to enter the cell for utilization [13].

Due to myocardial hypoxia and inflammatory response, the expression levels of DMT1 may be upregulated. This is because, in the hypoxic environment of AMI, the expression levels of hypoxia-inducible factor 1 alpha (HIF-1α) significantly increase. HIF-1α promotes the expression of the DMT1 gene by binding to the hypoxia-responsive element promoter region [14]. This process helps cells adapt to the hypoxic environment, maintaining iron uptake and utilization.

During AMI, the expression levels of FPN undergo significant changes. FPN is the only known cellular iron exporter. The expression levels of FPN are typically influenced by the regulation of hepcidin [15]. Hepcidin is a small peptide synthesized by the liver that binds to FPN, causing its endocytosis and degradation, thereby reducing FPN expression on the cell membrane [16]. Studies have shown that in AMI, due to inflammatory responses and hypoxic conditions, hepcidin expression significantly increases, which inhibits FPN function and reduces iron export [17]. During AMI, elevated levels of inflammatory mediators such as interleukin-6 (IL-6) [18] can also induce hepcidin expression. This further inhibits FPN, preventing the release of iron from cells into the plasma, leading to intracellular iron accumulation and increased oxidative stress in myocardial cells.

During AMI, transferrin, a key protein in iron metabolism, undergoes significant changes. Literature indicates that after AMI occurs, serum transferrin levels significantly decrease and are closely related to the extent of myocardial necrosis. This change may be associated with inflammatory responses and tissue damage [19]. The decrease in transferrin levels during the acute phase is usually accompanied by reductions in serum iron and total iron-binding capacity, while serum ferritin levels significantly increase [20]. These changes reflect the acute regulation of iron metabolism in the body, possibly due to the transport of iron from the blood to macrophages and other iron storage cells to address damage and repair processes. During AMI, the levels of transferrin receptor (TfR) and soluble transferrin receptor (sTfR) also show significant changes, reflecting the acute alterations in iron metabolism. sTfR is generated from the proteolytic cleavage of TfR on the cell membrane and is the secreted form of TfR1. Under conditions of iron deficiency or iron regulatory disorders, such as increased hepcidin inhibiting iron export, cells compensate for reduced iron uptake caused by low serum iron levels by increasing the expression of TfR1 to enhance the utilization of available serum iron. Therefore, elevated sTfR levels reflect increased iron demand, especially in the context of inflammation and disrupted iron metabolism [21]. Studies have found that sTfR levels significantly increase in AMI patients and are closely associated with the risk of future cardiovascular events [22]. Another study found that serum ferritin and sTfR levels were significantly elevated in patients with acute coronary syndrome, reflecting acute changes in iron metabolism.

**Figure 1:**
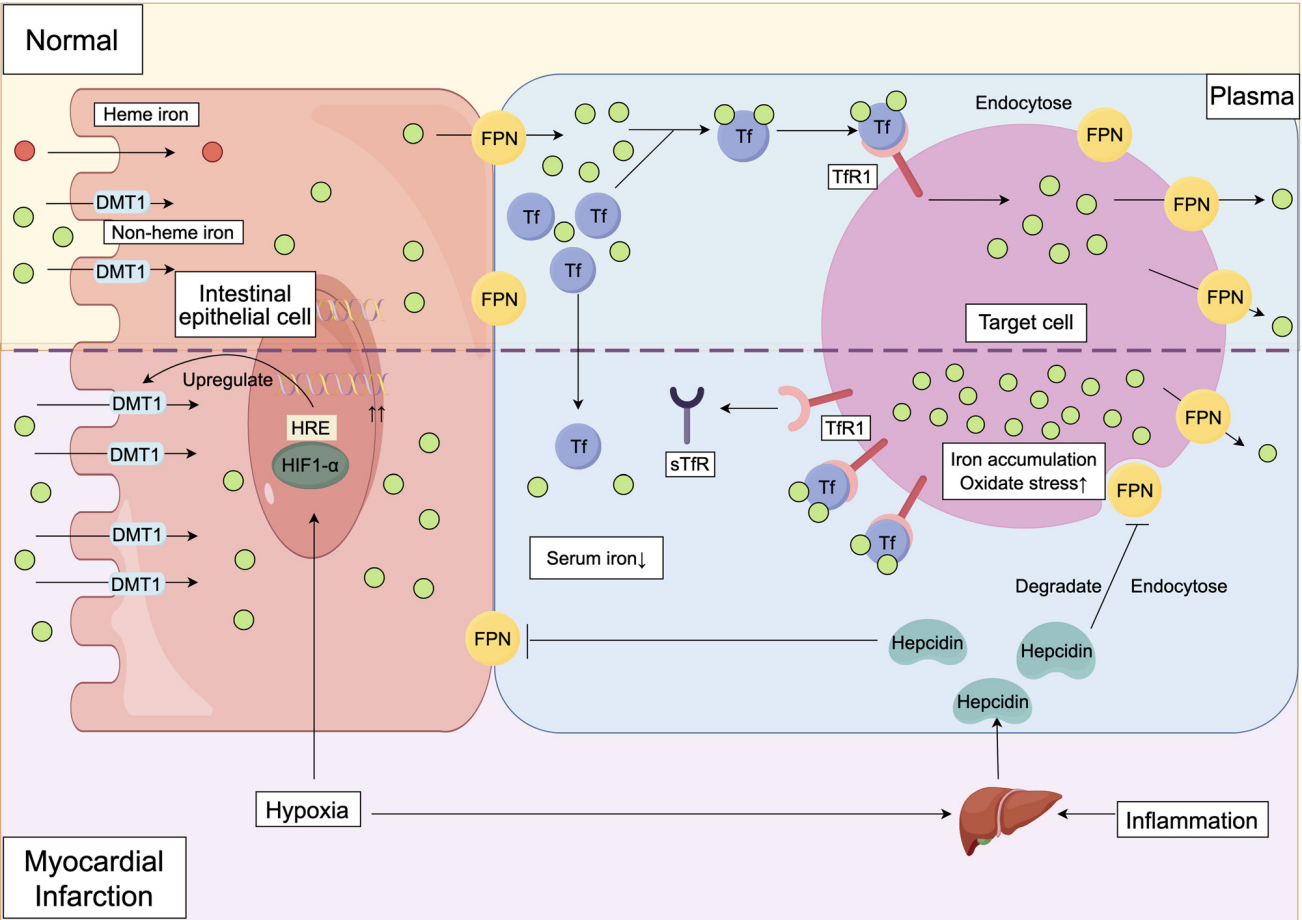
This diagram illustrates the impact of AMI on iron metabolism, including intestinal iron absorption, cellular iron transport, and oxidative stress. Under normal conditions, iron is primarily absorbed in the intestines, with non-heme iron being taken up by intestinal epithelial cells via DMT1 and exported into the bloodstream through FPN. In the plasma, iron binds to Tf, forming a transferrin–iron complex that enters target cells via TfR1. During AMI, the hypoxic environment activates hypoxia-inducible factor 1-alpha (HIF-1α), which upregulates DMT1 expression, enhancing intestinal iron absorption. At the same time, inflammatory responses stimulate the liver to produce hepcidin, which inhibits FPN expression, preventing the release of iron from cells into the bloodstream. This results in iron accumulation in cells, triggering oxidative stress and exacerbating myocardial injury. Additionally, AMI leads to a decrease in serum Tf levels, while TfR1 and sTfR levels increase, reflecting disrupted iron metabolism and increased demand for iron in myocardial cells. These pathological changes collectively contribute to increased oxidative stress and myocardial tissue damage after AMI, affecting the myocardial repair process.

In summary, the pathological alterations in iron metabolism during AMI – driven by increased iron absorption, impaired iron export, and abnormal protein levels – exacerbate oxidative stress and inflammatory responses, thereby creating complex effects on myocardial repair post-infarction (Figure 1).

## 3 The relationship and mechanisms between iron overload, ferroptosis, and VR after MI

Post-MI VR involves a complex adaptive response characterized by cardiomyocyte death, inflammation, fibrosis, and structural changes in the heart. Iron overload plays a critical role in this process, primarily by promoting oxidative stress and ferroptosis, which exacerbate myocardial injury and fibrosis.

### 3.1 Iron overload increases reactive oxygen species (ROS) production and exacerbates myocardial injury through Fenton reaction

Iron overload plays a pivotal role in myocardial injury and fibrosis, primarily by increasing the production of ROS, thereby triggering oxidative stress. ROS are highly reactive molecules, including superoxide anion ( \[{\text{O}}_{2}^{-}]\] ), hydroxyl radicals (˙OH), and hydrogen peroxide (H₂O₂). Under normal physiological conditions, the production and clearance of ROS are tightly regulated, maintaining cellular redox balance. However, in the context of iron overload, this balance is disrupted, leading to excessive accumulation of ROS. The Fenton reaction is central to this process, in which ferrous iron (Fe²⁺) reacts with H₂O₂ to generate highly reactive hydroxyl radicals (˙OH). These hydroxyl radicals cause extensive oxidative damage to key cellular components, including DNA, proteins, and membrane lipids.

The production of hydroxyl radicals not only induces lipid peroxidation of cell membranes, compromising membrane integrity but also leads to DNA strand breaks, causing irreversible damage and promoting apoptosis in cardiomyocytes. Additionally, lipid peroxidation products, such as malondialdehyde (MDA) and 4-hydroxynonenal, are highly toxic and further exacerbate myocardial cell injury and fibrosis.

Iron-induced oxidative stress also activates several pro-inflammatory signaling pathways, particularly the nuclear factor kappa-light-chain-enhancer of activated B cells (NF-κB) pathway. NF-κB is a crucial intracellular transcription factor that regulates the expression of multiple pro-inflammatory genes. Elevated oxidative stress activates NF-κB, causing its translocation into the nucleus, where it induces the overexpression of several pro-inflammatory cytokines, including tumor necrosis factor-alpha, IL-6, and interleukin-1 beta. These cytokines not only amplify local inflammatory responses but also promote the remodeling of the extracellular matrix in the myocardium, driving myocardial fibrosis and the progression of VR.

Moreover, ROS generated through the Fenton reaction can activate stress kinase pathways, such as the p38 mitogen-activated protein kinase (MAPK) and c-Jun N-terminal kinase (JNK) pathways. Activation of these stress kinases is closely linked to apoptosis and fibrosis in cardiomyocytes. Studies have shown that sustained activation of p38 MAPK and JNK pathways enhances myocardial apoptosis and inflammation, accelerating pathological remodeling of the myocardial tissue.

Thus, iron overload, through multiple mechanisms, promotes myocardial cell injury and inflammatory responses. It not only directly damages the structural integrity of cardiomyocytes but also exacerbates inflammatory signaling, leading to fibrosis and VR. Evidence from clinical and experimental studies further supports the role of iron overload in adverse cardiac remodeling. In the study by Bulluck et al., the presence of residual myocardial iron following intramyocardial hemorrhage (IMH) during the convalescent phase of re-perfused STEMI was investigated. The study found that residual myocardial iron was associated with adverse left VR. This suggests that residual iron deposition post-IMH contributes to structural changes and functional deterioration of the left ventricle [23]. In a study using a C57BL/6J mouse model, MI was induced by permanent left anterior descending artery ligation, and the role of iron in VR was explored. The study found that these mice exhibited left ventricular (LV) dilation and LV systolic dysfunction, along with cardiomyocyte hypertrophy and interstitial fibrosis in the remote region. Conversely, dietary iron restriction alleviated LV dilation and systolic dysfunction, while also reducing cardiomyocyte hypertrophy and interstitial fibrosis [24].

### 3.2 Ferroptosis as a key driver of cellular and tissue damage in post-MI remodeling

Iron-mediated oxidative stress can activate ferroptosis, an iron-dependent programmed cell death mechanism, further aggravating myocardial injury. Unlike traditional apoptosis and necrosis, ferroptosis is mainly caused by lipid peroxidation, which disrupts the structure of the cell membrane. In the process of ferroptosis, iron-catalyzed peroxidation of polyunsaturated fatty acids (PUFAs) is the core mechanism. This lipid peroxidation causes irreversible damage to the cell membrane, compromising its integrity and ultimately leading to cell death. PUFAs, as key components of cell membranes, are highly susceptible to oxidation, particularly in cases of iron overload. In such conditions, iron generates large amounts of ROS through Fenton or Haber–Weiss reactions, further promoting lipid oxidative damage.

During this process, glutathione peroxidase 4 (GPX4) and solute carrier family 7 member 11 (SLC7A11) are key regulatory molecules [25]. GPX4 reduces phospholipid hydroperoxides, thereby inhibiting lipid peroxidation and preventing ferroptosis. SLC7A11, as a cystine-glutamate antiporter, is responsible for the uptake of cystine, which is a precursor for glutathione (GSH) synthesis. GSH is a critical cofactor for GPX4 activity. Downregulation of SLC7A11 reduces intracellular GSH levels, weakening the antioxidant function of GPX4. When GPX4 activity is inhibited or inactivated, lipid peroxides are not efficiently reduced, leading to exacerbated PUFA peroxidation and triggering ferroptosis.

Following MI, local hypoxia, inflammatory responses, and reperfusion injury lead to iron accumulation within cardiomyocytes, which increases ROS production and triggers ferroptosis. This iron-dependent cell death mechanism has been identified as a key contributor to myocardial injury and subsequent structural alterations. For instance, Miyamoto et al. demonstrated that iron overload, via heme degradation in the endoplasmic reticulum, triggers ferroptosis during the ischemia–reperfusion (I/R) process. The resulting ferroptosis leads to increased lipid peroxidation and oxidative stress, which not only accelerates cardiomyocyte death but also promotes adverse cardiac remodeling, such as LV dilation and systolic dysfunction [26]. Similarly, in a study by Baba et al., using an I/R mouse model, excess iron was found to induce ferroptosis and oxidative stress, resulting in cardiomyocyte damage and adverse changes in cardiac structure. Interestingly, activation of the mechanistic target of rapamycin was shown to mitigate these harmful effects by reducing cardiomyocyte death and preventing structural deterioration of the heart [27].

## 4 The role of iron deficiency in post-MI VR

Iron homeostasis plays a dual role in cardiovascular health, particularly in the context of VR after MI. While iron overload promotes oxidative stress and exacerbates cardiomyocyte injury through ferroptosis, iron deficiency also contributes to adverse cardiac remodeling via different molecular mechanisms.

First, iron is a crucial cofactor for mitochondrial function and energy metabolism. After AMI, cardiomyocytes are damaged and require a large amount of energy for repair and regeneration. However, iron deficiency leads to a decrease in mitochondrial oxidative phosphorylation, resulting in insufficient cellular energy production, which limits the repair process of cardiomyocytes. This energy metabolic insufficiency not only affects the survival of cardiomyocytes but also delays the repair of damaged myocardium, causing abnormal VR and subsequently affecting cardiac function. This process is related to the impact of iron deficiency on the electron transport chain within mitochondria, as iron deficiency weakens ATP production, ultimately leading to cellular dysfunction [28].

Second, iron deficiency adversely affects myocardial performance by impacting the expression and function of key proteins involved in oxygen transport and utilization, such as myoglobin and cytochrome c oxidase (COX). Myoglobin is an oxygen-binding protein found in cardiac and skeletal muscles, responsible for storing and transporting oxygen within tissues. When myoglobin levels decrease, the tolerance of cardiomyocytes to hypoxia is reduced, especially during acute ischemia or after MI, further exacerbating the hypoxic state under iron deficiency. COX is a key enzyme in the mitochondrial respiratory chain, catalyzing the reduction of oxygen and completing the final step of the electron transport chain, directly influencing ATP production. Studies have shown that iron deficiency inhibits COX activity, leading to reduced ATP generation, which compromises the energy supply and contractile function of cardiomyocytes. These changes further weaken myocardial metabolism and function, worsening the prognosis of patients with heart failure or after MI. Alnuwaysir et al. pointed out that iron deficiency not only impairs myocardial function by reducing ATP production but also affects other key proteins involved in oxygen transport and mitochondrial respiration, thereby exacerbating myocardial damage [29]. This impact is evident not only in chronic heart failure but also during the repair process following acute myocardial injury. Further research shows that iron deficiency inhibits the activity of COX, hindering the myocardium’s aerobic metabolic capacity, leading to abnormal energy metabolism, and ultimately affecting cardiac function [30]. This reduction in energy supply negatively impacts the heart’s long-term metabolic adaptability, worsening VR and fibrosis progression.

Furthermore, iron deficiency exacerbates the inflammatory response by activating key pro-inflammatory pathways such as the p38 MAPK–NF-κB pathway. Studies have shown that iron deficiency significantly enhances NF-κB activity in macrophages, with NF-κB being a central transcription factor for various pro-inflammatory cytokines [31]. In iron-deficient conditions, the phosphorylation levels of p38 MAPK increase, which subsequently activates the NF-κB signaling pathway, leading to increased expression of pro-inflammatory factors such as matrix metalloproteinase-9 and extracellular matrix metalloproteinase inducer. These factors play a critical role in myocardial fibrosis and VR [32].

The effects of iron deficiency on cardiac remodeling have been supported by various studies. A study by Huang et al. found that lower serum iron concentrations, independent of hemoglobin levels, were associated with the higher thrombolysis in myocardial infarction scores and poorer LV performance 6 months after primary angioplasty in patients with AMI. This highlights the potential role of serum iron as a prognostic marker for LV function following angioplasty [33]. In another clinical study involving 306 patients with chronic heart failure, it was found that those with iron deficiency exhibited significant left heart structural abnormalities. These included left atrial and LV dilation, hypertrophy of the inter-ventricular septum and left ventricular posterior wall thickness, and an increased left ventricular mass index. These findings suggest that iron deficiency adversely impacts cardiac remodeling in patients with chronic heart failure [34].

## 5 The association between iron ion levels and aneurysm formation after AMI

The development of left ventricular aneurysm (LVA) is a serious and well-known complication following AMI [35]. LVA arises from the pathological dilation and thinning of the ventricular wall at the site of infarction. The extensive cardiomyocyte necrosis, inflammatory responses, and ischemia during AMI alters the structural integrity of the myocardium, eventually leading to aneurysmal changes in the affected area. This structural deformation weakens the heart’s pumping ability, increasing the risk of heart failure, arrhythmias, and sudden death.

Iron overload, especially elevated serum iron levels, exacerbates oxidative stress and inflammation, promoting myocardial damage and fibrosis, both of which are linked to a higher incidence of LVA formation. For instance, a study by Feng et al. indicated that impaired renal function and abnormal ferritin levels are independent risk factors for LVA formation after AMI. This hospital-based case–control study showed that a glomerular filtration rate below 60 mL/min and abnormal ferritin levels significantly increased the incidence of LVA [36]. Similarly, a study by Behrouzi et al. demonstrated that the iron chelator deferiprone (DFP) significantly reduced myocardial fibrosis in a porcine model of AMI. The treatment accelerated the resolution of hemorrhage and reduced oxidative stress and inflammation, which are key drivers of adverse myocardial remodeling and fibrosis, potentially lowering the rates of LVA formation [37].

Not only does iron overload exacerbate oxidative stress and inflammation, contributing to myocardial damage and fibrosis, but iron deficiency also impairs the energy metabolism of cardiomyocytes. In conditions of iron deficiency, the reduced availability of iron leads to a decrease in the metabolic capacity of cardiomyocytes, increasing their susceptibility to hypoxic damage and fibrosis. This, in turn, may contribute to the formation of LVA following MI [38]. Specifically, iron deficiency disrupts the synthesis of mitochondrial iron-sulfur cluster proteins and cytochromes, both of which are crucial for proper oxidative phosphorylation. This impairment of oxidative phosphorylation compromises the energy production necessary for cellular repair and function, exacerbating myocardial injury and remodeling [39]. Although there is currently no direct evidence linking iron deficiency with the formation of LVA after MI, studies have demonstrated a strong association between iron deficiency and aneurysm formation. For example, research by Li et al. showed that iron deficiency promotes the degeneration of the aortic media by disrupting the cytoskeleton of vascular smooth muscle cells, leading to the development of aortic aneurysms. This mechanism highlights the detrimental effect of iron deficiency on vascular stability, suggesting that it may play a similar role in the process of left VR following MI [40]. In addition, a case report describing profound iron deficiency anemia in a child with irreversible dilated cardiomyopathy provides further evidence of the severe consequences of iron deficiency on cardiac structure and function. Despite correction of the anemia, the patient continued to experience progressive heart failure, highlighting that iron deficiency may result in irreversible myocardial damage in some cases. This case underscores the importance of timely management of iron deficiency to prevent permanent cardiac remodeling and dysfunction [41].

## 6 Therapeutic strategies for iron metabolism regulation to prevent post-MI VR and aneurysm formation

Ventricular remodeling and aneurysm formation after AMI significantly affect patient prognosis. Studies have shown that iron metabolism plays a crucial role in these processes. Regulating iron metabolism to prevent and treat post-AMI VR and aneurysm formation has become an important research direction (Table 1).

**Table 1:** Therapeutic approaches for regulating iron metabolism in preventing post-MI VR

Therapeutic strategy	Mechanism of action	Effect on post-MI remodeling	References
Iron chelators (e.g., deferoxamine, DFP)	Reduces iron overload, inhibits oxidative stress and inflammation	Lowers fibrosis, reduces VR and aneurysm formation	[42–44]
Hepcidin/ferroportin regulation	Inhibits hepcidin or enhances FPN to reduce intracellular iron levels	Reduces oxidative stress and prevents adverse cardiac remodeling	[17]
Ferroptosis inhibitors (e.g., ferrostatin-1, liproxstatin-1)	Reduces lipid peroxidation, increases antioxidant levels (GPX4, GSH)	Mitigates myocardial injury and improves heart function after MI	[45,46]
Iron supplementation	Enhances energy metabolism and reduces hypoxia-induced damage in cardiomyocytes	Improves myocardial function in iron-deficient patients, reduces fibrosis	[47,48]
Gene and targeted therapies	Modulates iron-regulatory genes (e.g., HIF-2α, FPN) to reduce oxidative stress	Offers potential for long-term reduction of iron overload and cardiac fibrosis	[49,50]

Abbreviations: FPN, ferroportin; GPX4, glutathione peroxidase 4; GSH, glutathione; HIF-2α, hypoxia-inducible factor 2 alpha; MI, myocardial infarction.

Iron chelators have shown significant effects in reducing iron overload. Iron overload promotes oxidative stress and inflammatory responses, exacerbating myocardial damage and fibrosis, thereby contributing to aneurysm formation. Deferoxamine decreases intracellular iron levels in cardiomyocytes, reducing the production of ROS and thereby mitigating oxidative stress and inflammation [42]. Additionally, iron chelators can reduce myocardial damage and fibrosis by inhibiting the NF-κB signaling pathway, which decreases the release of inflammatory cytokines [43]. Recent studies suggest that the iron chelator DFP not only reduces iron overload but also offers protective effects against adverse cardiac remodeling following MI. In canine models of reperfused MI with IMH, DFP has been shown to significantly reduce iron deposition within hemorrhagic infarcted areas. This reduction in iron levels subsequently decreases fat infiltration in the affected regions, leading to improved LV function and mitigating the progression of VR by limiting inflammation and lipid peroxidation within the myocardial tissue [44].

Regulating hepcidin and FPN is another effective strategy. Hepcidin binds to FPN, causing its endocytosis and degradation, which reduces FPN expression on the cell membrane and thus decreases iron export. In the hypoxic and inflammatory environment of AMI, hepcidin expression significantly increases, leading to iron accumulation and oxidative stress in cardiomyocytes. By inhibiting hepcidin or enhancing FPN function, intracellular iron levels in cardiomyocytes can be reduced, thereby decreasing oxidative stress and inflammation and preventing VR and aneurysm formation [17].

Ferroptosis inhibitors, such as ferrostatin-1 and liproxstatin-1, have been shown to significantly reduce oxidative stress by decreasing lipid peroxidation and regulating GPX4 activity. These inhibitors have demonstrated effectiveness in reducing cardiomyocyte death and VR. In a study examining the effects of ferrostatin-1 on cardiomyocyte ferroptosis post-MI, a myocardial infarction murine model was used, which was created by ligating the left anterior descending coronary artery in adult male C57BL/6J mice. It was demonstrated that ferrostatin-1 significantly suppressed ferroptosis through the activation of Nrf2 signaling. The study found that ferrostatin-1 not only decreased the levels of redox-active iron and lipid peroxidation markers such as MDA but also increased antioxidant levels such as GSH and superoxide dismutase. These effects collectively led to a reduction in myocardial injury and mitigated VR, thereby improving heart function [45]. Feng et al. investigated the protective effects of liproxstatin-1, a ferroptosis inhibitor, on I/R injury in the myocardium using a mouse model. Specifically, the study utilized adult male C57BL/6J mice (9–12 weeks old), and myocardial I/R was induced in isolated perfused hearts using the Langendorff perfusion system. The study demonstrated that liproxstatin-1 reduced myocardial infarct size by decreasing VDAC1 expression and oligomerization, restoring GPX4 levels, and reducing mitochondrial ROS production. Although liproxstatin-1 did not affect calcium-induced mitochondrial permeability transition pore opening, its cardioprotective effects were primarily mediated through the inhibition of ferroptosis-related pathways [46].

Iron deficiency can also lead to impaired energy metabolism in cardiomyocytes, increasing the risk of myocardial damage and fibrosis. Studies have shown that iron supplementation can improve myocardial function in iron-deficient patients and reduce the risk of fibrosis. By regulating metabolic pathways in cardiomyocytes, iron supplements not only enhance energy supply but also reduce hypoxia and oxidative stress-related damage to cardiomyocytes [47]. A retrospective study on diabetic patients undergoing surgical revascularization found that iron deficiency was associated with impaired recovery of LV function. The study showed that iron supplementation helped improve myocardial function and reduced the risk of fibrosis and adverse remodeling in these patients [48].

Gene therapy and targeted therapy offer new possibilities for regulating iron metabolism. By modulating the expression of iron metabolism-related genes, such as hypoxia-inducible factor-2 alpha (HIF-2α) and FPN, oxidative stress and inflammatory responses can be reduced, thereby protecting cardiomyocytes [49]. For instance, HIF-2α knockout mouse models have shown that HIF-2α upregulates FPN in hypoxic environments, increasing iron absorption and accumulation, which can exacerbate oxidative stress and fibrosis [50]. Targeting the regulation of these gene expressions can reduce excessive iron accumulation, lowering the risk of myocardial damage and fibrosis. Future research can further explore the specific regulatory mechanisms of these genes and proteins, providing a theoretical basis for gene therapy and targeted therapy.

## 7 Summary

Ventricular remodeling and LVA formation are critical complications following AMI, significantly impacting patient prognosis. Iron metabolism plays a dual role in these processes, with both iron overload and deficiency contributing to adverse cardiac outcomes. Iron overload exacerbates myocardial damage by promoting oxidative stress, inflammation, and ferroptosis, a form of iron-dependent cell death, leading to ventricular dilation and fibrosis. On the other hand, iron deficiency impairs mitochondrial function, energy metabolism, and cardiomyocyte repair, further aggravating VR and predisposing the myocardium to hypoxic damage. Therapeutic strategies targeting iron metabolism, such as the use of iron chelators, ferroptosis inhibitors, and iron supplementation, hold promise in mitigating the detrimental effects of iron dysregulation. These approaches aim to balance iron levels, reduce oxidative stress, and promote cardiomyocyte survival and function. Gene therapy and modulation of iron-regulatory proteins, such as hepcidin and FPN, offer novel avenues for preventing maladaptive remodeling post-AMI. Future research should focus on elucidating the precise molecular mechanisms linking iron metabolism with myocardial remodeling and aneurysm formation to develop targeted therapies that improve long-term cardiac outcomes.
